# Hypouricaemic and nephroprotective effects of *Poria cocos* in hyperuricemic mice by up-regulating ATP-binding cassette super-family G member 2

**DOI:** 10.1080/13880209.2021.1885450

**Published:** 2021-03-02

**Authors:** Danling Liang, Tianqiao Yong, Xue Diao, Shaodan Chen, Diling Chen, Chun Xiao, Dan Zuo, Yizhen Xie, Xinxin Zhou, Huiping Hu

**Affiliations:** a Guangdong Provincial Key Laboratory of Microbial Safety and Health and State Key Laboratory of Applied Microbiology Southern China, Guangdong Institute of Microbiology, Guangdong Academy of Sciences, Guangzhou, China; b Guangdong Yuewei Edible Fungi Technology Co., Guangzhou, China; c School of Pharmaceutical Science, Guangzhou University of Chinese Medicine, Guangzhou, China; d Affiliated Hospital of Guangdong Medical University, Zhanjiang, China; e Guangzhou Institutes of Biomedicine and Health, Chinese Academy of Sciences, Guangzhou, China

**Keywords:** Uric acid, transportation, bioactives, higher fugal, molecular docking

## Abstract

**Context:**

*Poria coco* F.A.Wolf (Polyporaceae) dispels dampness and promotes diuresis implying hypouricaemic action.

**Objective:**

To examine hypouricaemic action of *Poria coco*.

**Materials and methods:**

Ethanol extract (PCE) was prepared by extracting the sclerotium of *P. cocos* with ethanol, and the water extract (PCW) was produced by bathing the remains with water. PCE and PCW (50, 100 and 200 mg/kg, respectively) were orally administered to hyperuricemic Kunming mice (*n* = 8) to examine its hypouricaemic effect. Also, molecular docking was performed.

**Results:**

*P. cocos* showed excellent hypouricaemic action, decreasing the serum uric acid of hyperuricaemia (HUA) control (526 ± 112 μmol/L) to 178 ± 53, 153 ± 57 and 151 ± 62 μmol/L (*p* < 0.01) by PCE and 69 ± 23, 63 ± 15 and 62 ± 20 μmol/L (*p* < 0.01) by PCW, respectively. According to SCrs, BUNs and H&E staining, PCE and PCW partially attenuated renal dysfunction caused by HUA. They presented no negative effects on ALT, AST and ALP activities. They elevated ABCG2 (ATP-binding cassette super-family G member 2) mRNA and protein expression in comparison to HUA control. In molecular docking, compound **267**, **277**, **13824**, **15730** and **5759** were predicted as the top bioactives of *P. cocos* against HUA, which even presented better scores than the positive compound, oestrone 3-sulfate.

**Discussion and conclusions:**

This paper demonstrated the hypouricaemic and nephroprotective effects of *P. cocos* in hyperuricemic mice by up-regulating ABCG2. These results may be useful for the development of a hypouricaemic agent.

## Introduction

Over the past decades, the prevalence of hyperuricaemia (HUA) (Song et al. 2018; Chen et al. 2019) and its directly induced gout (Kuwata et al. 2017; Xu et al. 2017; Barkas and Elisaf 2018) have been rising (Kuo et al. 2015; Liu et al. 2015; Rai et al. 2017). Clinically, HUA patients require long-term urate-lowering managements or even life-long treatment (Janssen et al. 2018). Surprisingly, only a few drugs are approved by U.S. Food and Drug Administration, including allopurinol, benzbromarone and lesinurad. Unfortunately, they are accompanied with considerable side effects, such as Steven Johnson syndrome (allopurinol) (Yang CY et al. 2015), severe liver toxicity (benzbromarone) (Lee et al. 2008) and even severe renal toxicity (lesinurad), which rendered them unsuitable for long-term usage. Hence, developing novel and safe therapies against HUA are urgently needed.

Currently, many scholars are focussing on herb (Hu et al. 2020) or even higher fungi therapies (Chang et al. 2015). In this regard, traditional Chinese medicine (TCM) is attractive since the medicines recorded have been exploited usefully for thousands of years. Also, they have been classified systematically into several categories according to the clinical practices of doctors. Most are effective, except the ill-defined ones for maintaining secrecy and protecting intellectual property in ancient years. According to TCM theory, the modern concept of HUA should be recognized as ‘damp toxin’ and ‘Lijie’, corresponding to its pathology and symptom, respectively (Bo and Cao 2008; Qiu 2008). ‘Damp toxin’ means that HUA were induced by long-term exposures to high- or low-temperature or even to too cold or hot wind. ‘Lijie’ refers to the allergic inflammatory pains at ankle or other joints caused by HUA or gout, which resembles the status of biting by a tiger. Accordingly, HUA was classified into four stages (Kong et al. 2000): (1) dampness-heat obstruction, characterized with acute joint pain and dribbling urination; (2) blood stasis in meridians, featured with ankylosis and arthralgia at extremities; (3) insufficiency of Qi (power or function) in kidney, displaying as oedema, dizziness, soreness and weakness of waist and knees, which may be ascribed to uric acid nephrolithiasis; (4) dual deficiencies in Qi and Yin, resulting in feeling heavy muscle joints, difficulty urinating and stiffness of muscle and joint. The four are induced by a long-term HUA for 1 and 2 or an asymptomatic HUA for 3 and 4 correspondingly. According to this theory, TCMs against HUA should eliminate dampness, promote diuresis and invigorate the circulation of blood and functions of kidney and spleen (Yan et al. 2016).

*Poria coco* F.A.Wolf (Polyporaceae), also called Fuling in China, identified by a mycology authority Professor Huiping Hu in this work, has a long history as a Chinese traditional fungal medicine for eliminating dampness, promoting diuresis, invigorating the spleen and tranquilizing the mind. Moreover, *P. cocos* played a role in the Wuling San, a hypouricaemic TCM formula (Yang Y et al. 2015). Also, many triterpenes and polysaccharides have been isolated from *P. cocos*, exerting bioactivities including antitumor (Chen et al. 2009; Lee et al. 2018), antioxidant (Tang et al. 2014), antirejection (Zhang et al. 2004), anti-hyperlipidaemia, anti-hyperglycaemia (Li T et al. 2011), nematicide (Li et al. 2005), antibacteria (Wang et al. 2018) and anti-inflammation (Lee S et al. 2017; Lee SR et al. 2017). However, there is no report about its hypouricaemic effect.

In this paper, we investigated the hypouricaemic action of *P. cocos* in hyperuricemic mice. In order to examine its influence on inner organs, inner organ coefficients were recorded. Also, haematoxylin–eosin (H&E) staining was used to examine the liver and renal morphological alterations. Besides, reverse transcription PCR (RT-PCR) and Western blotting were conducted to inspect the modulated genes by *P. cocos*. Finally, molecular docking was used for screening the potential compounds in *P. cocos* against HUA. This work may provide a clue for developing a new phytomedicine against HUA.

## Materials and methods

### Reagents and materials

Potassium oxonate (PO, 98.0%), hypoxanthine (HX, 99%) and benzbromarone (98%) were purchased from Aladdin Reagent Co. (Shanghai, China). Allopurinol (98%) was obtained from Tokyo Chemical Industry Co. (Tokyo, Japan). Assay kits for uric acid, blood urea nitrogen (BUN) and creatinine (CREA) measurements and alanine aminotransferase (ALT), aspartate aminotransferase (AST) and alkaline phosphatase (ALP) activity measurements were obtained from Mindray Medical Corp. (Shenzhen, China). TRIZOL reagent was acquired from Invitrogen Corp. (Carlsbad, CA). PCR primers were obtained from Sangon Biotech Co., Ltd. (Shanghai, China). RevertAid™ First Strand cDNA Synthesis Kit was bought from Thermo Fisher Scientific Inc. (Vilnius, Lithuania). SYBR Premix Ex Taq II was offered by Takara Bio Inc. (Otsu, Japan). Antibodies against ABCG2, OAT1, OAT3 and OCT2 were supplied by Affinity Biosciences (Cincinnati, OH). Rabbit GAPDH antibody and secondary HRP-conjugated goat anti-rabbit IgG were purchased from ProteinTech Group (Chicago, IL).

### Medicinal fugal extracts

*P. cocos* was supplied by Guangdong Yuewei Edible Fungi Com. (Guangzhou, China) and identified by a mycology authority Professor Huiping Hu. A voucher specimen (no. YW20181108-PC) was stored at Guangdong Institute of Microbiology. One hundred grams *P. cocos* was immersed with 2 L of ethanol at 65 °C for 3 h for three times. Then, the acquired extract was filtered and evaporated to yield ethanol extract (PCE, 1.22 g; 1.22%). Water extract (PCW; 1.29 g; 1.29%) was obtained by extracting the remains using 2 L water as solvent at 85 °C for 3 h for three times, followed by lyophilization. The HPLC fingerprints (Supplementary Figures S1 and S2) of PCE and PCW were provided and a control of standard chemical compound (pachymic acid, Supplementary Figure S3) was utilized for identifying *P. cocos*.

### Animals

Guangdong Institute of Microbiology approved (ID: GT-IACUC20180426-1, 26 April 2018, Guangzhou, China) all animal experimental protocols in this study. Male specific pathogen-free (SPF) Kunming mice (20 ± 2 g) were provided by the Guangdong Provincial Medical Laboratory Animal Centre (Guangzhou, China) and were housed in laboratory conditions with free water and feed for a week before the experiment. Temperature was maintained between 24 and 26 °C. Preliminarily, mice were randomized into 10 groups (*n* = 8): normal control, HUA control, allopurinol and benzbromarone controls and drug groups with PCE and PCW at doses of 50, 100 and 200 mg/kg, respectively. The hyperuricemic mice were established by a method reported in our previous studies (Liang et al. 2018), which was used conventionally as standard animal models. Briefly, 1 h before the drug administration, mice were treated with HX orally at a dose of 500 mg/kg as well as with PO intraperitoneally at a dose of 300 mg/kg for model establishment. Allopurinol and benzbromarone served as positive controls. The normal controls were injected and administrated with the same volume of physiological saline (0.9%) as the HUA control.

### Drug administration

Mice in each treatment were treated once a day for a week. The allopurinol and benzbromarone controls were medicated intragastrically with allopurinol (5 mg/kg) and benzbromarone (7.8 mg/kg), correspondingly. For PCE and PCW groups, mice were administrated orally with PCE and PCW respectively at three doses of 50, 100 and 200 mg/kg which we selected based on a preliminary experiment. The normal and HUA controls were treated orally with the same volume of physiological saline (0.9%).

### Determination of uric acid, BUN, SCr and ALT, AST, ALP activities

Urine was collected when urination occurred when we pressed the bladder slightly on the 7th day of the animal experiment. Then, blood was collected when mice were sacrificed. To obtain serum, the blood was centrifuged at 3800 rpm for 10 min at 4 °C, and the supernatant was separated and then stored at −20 °C. The serum was applied for determination of blood uric acid (SUA), BUN, serum creatinine (SCr) levels and ALT, AST and ALP activities. Urine was also used to measure the excretory uric acid. Liver, renal, thymus, spleen and small intestine tissues were excised, weighted, recorded and then strored at −80 °C. A part of liver and kidney were immersed in neutral 10% formalin immediately. SUA, urine uric acid (UUA), BUN, SCr, ALT, AST and ALP were measured by exploiting BS-480 Mindray Automatic Clinical Blood Chemistry Analyzer (Mindray Medical Corp., Shenzhen, China).

### Organ coefficients

Organ coefficients, including liver, kidney, spleen as well as thymus, represented as tissue weighting factor, were calculated by dividing the weight of organ by the corresponding 7th day body weight of individual mouse.

### Pathological histology

Renal and liver tissues of mice were fixed in neutral 10% formalin and then dehydrated gradually by increasing the concentrations of ethanol. The kidney tissues were then embedded in paraffin, sectioned at 5 μm and prepared for staining with H&E. The treated sections were visualized under light microscopy (Nikon, Tokyo, Japan) at ×200 magnification.

### RT-PCR analysis

The total RNAs in kidney and small intestine were extracted by using TRIzol reagent according to a procedure of homogenization, centrifugation, processing with chloroform, isopropanol and washing with ethanol (75%) according to the manufactory’s instructions. Then, the total RNA (2 μg) was added to each of the tubes, together with oligo (dT) 18 primer (1 μL), 5 × reaction buffer (4 μL), dNTPs (2 μL), RNA inhibitor (1 μL), M-MLV reverse transcriptase (1 μL) and the tube volumes were adjusted to 20 μL using DEPC water without RNase. The tubes were kept at 42 °C for 60 min, and then the reactions were terminated by heating the RNA solution at 70 °C for 5 min. The obtained cDNA was diluted with double distilled (dd) water, and PCR amplification was performed using primers at appropriate conditions (Table 1). Tubes containing 2 × SYER Premix Ex TapTMII (10.0 μL), forward primer (0.4 μL), reverse primer (0.4 μL), 50 × ROX Reference Dye (0.2 μL) and cDNA (2 μL) were adjusted to a volume of 20 μL with dd water. PCR was performed with an initial heat denaturation at 95 °C for 30 s, and the PCR cycles were repeated 40 times under the following conditions: denaturation at 95 °C for 5 s, annealing at 60 °C for 60 s. GAPDH was used as an endogenous standard.

**Table 1.** PCR primers and RT-PCR cycles for the key genes associated to hyperuricaemia.

Description	GenBank	Primer name	Primer sequences (5′–3′)	Product size (bp)	*T*_m_ (°C)	Thermal cycles
GAPDH^a^	NM_001289726.1	M-GAPDH-S	AGGTCGGTGTGAACGGATTTG	123	60	40
		M-GAPDH-A	TGTAGACCATGTAGTTGAGGTCA			
ABCG2^b^	NM_001355477.1	M-ABCG2-S	CACTGACCCTTCCATCCTCTTC	103	60	40
		M-ABCG2-A	GCCCTGTTTAGACATCCTTTTCA			
OAT1^c^	NM_008766.3	M-SLC22A6-S	CACCTGCTAATGCCAACCTC	109	60	40
		M-SLC22A6-A	CCATTGTGCGGGAAAGGAAA			
OAT3^d^	NM_001164635.1	M-SLC22A8 -S	CTGCCTTCTTCATCTTCTCCTTG	135	60	40
		M-SLC22A8-A	CTTCCTCCTTCTTGCCGTTG			
OCT2^e^	NM_013667.3	M-SLC22A2 -S	CACAACCCAACCTCACTTACC	81	60	40
	NM_013667.3	M-SLC22A2-A	CATCAGTGCAACAAACTGGGC			
GLUT9^f^	NM_001012363.2	M-SLC2A9-S	CCTCCTTCCTGTGGACTCTG	173	60	40
		M-SLC2A9-A	TCTTTGTCCTCCTCTGCTGG			
URAT1^g^	NM_009203.3	M-SLC22A12-S	CGCTTCCGACAACCTCAATG	254	60	40
		M-SLC22A12-A	CTTCTGCGCCCAAACCTATCT			

^a^ Glyceraldehyde-3-phosphate dehydrogenase.

^b^ ATP-binding cassette super-family G member 2.

^c^ Organic anion transporter 1.

^d^ Organic anion transporter 3.

^e^ Organic cation transporter 2.

^f^ Glucose transporter 9.

^g^ Uric acid transporter 1.

### Western blot analysis

Kidney samples were homogenized with 10 equivalent volumes of RIPA lysis buffer, supplemented with PMSF in an ice bath for 30 min, and then centrifuged (12,000 rpm, 10 min, 4 °C) to extract the total protein adequately. An equivalent of 5 μL protein samples were separated by 10% SDS-PAGE and then transferred onto a PVDF membrane (Millipore, Burlington, MA). The non-specific binding sites of the membranes were blocked with 5% skimmed milk in TBST (Tris-buffered saline with 0.1% Tween-20). Then, the membranes were probed overnight with specific primary antibodies diluted in TBST: ABCG2 (800:1), OAT1 (1000:1), OAT3 (800:1), OCT2 (1000:1) and GAPDH (20,000:1), followed by secondary HRP-conjugated goat anti-rabbit IgG (Immunoglobulin G, 6000:1) antibody for 1 h. Eventually, the membranes were mixed with ECL (Enhanced Chemiluminescence, Servicebio Co., Wuhan, China) and exposed to immunoreactive bands by Tanon 5200Muti system (Tanon Science and Technology Co., Ltd., Shanghai, China). All blots were repeated at least three times. The optical density of each blot band was analysed by Image J software (NIH, Bethesda, MD).

### Statistical analysis

The statistical analysis was processed with the professional data-processing program SPSS (Release 17.0, 2001, IBM SPSS Inc., Chicago, IL). All data were expressed as mean ± standard deviation (SD) and analysed by one-way analysis of variance (ANOVA). All statistics are presented with GraphPad Prism 7 (GraphPad Software, Inc., San Diego, CA), and the difference was considered statistically significant when *p* < 0.05 or *p* < 0.01 as compared by the two-tailed Student’s *t-*test.

### Virtual screening of bioactives via molecular docking

*P. cocos* compound database was built by collecting compounds of *P. cocos* from TCMID (Xue et al. 2013), TCMSP (Ru et al. 2014) and literatures via TCMAnalyzer Web Service (Liu et al. 2018). ABGG2 (Jackson et al. 2018) was chosen as receptor (PDB ID: 6FEQ). Active pocket was defined as a sphere of 10 Å in diameter with the centre at D6T. CDOCKER (Gagnon et al. 2016) was used to dock the compounds into the active pocket. Top ranked compounds were picked up for further analysis.

## Results

### Biochemical parameters suggested remarkable hypouricaemic, nephroprotective and liver protective effects of *P. cocos* extracts

First, we established the hyperuricemic models (526 ± 112 μmol/L, *p* < 0.01) successfully by dosing PO and HX to normal mice (71 ± 13 μmol/L, Table 2), which elevated SUAs remarkably. Moreover, declines of SUAs caused by positive drugs, allopurinol (296 ± 96 μmol/L, *p* < 0.01) and benzbromarone (258 ± 65 μmol/L, *p* < 0.01), were observed significantly, which further established that the models were established successfully. It was important that significant reductions of SUAs were induced by PCE and PCW at various doses in hyperuricemic mice. In detail, PCE at doses of 50, 100 and 200 mg/kg decreased the SUAs of hyperuricemic mice to 178 ± 53, 153 ± 57 and 151 ± 62 μmol/L (*p* < 0.01). Meanwhile, PCW at the same doses descended the SUAs of hyperuricemic mice to 69 ± 23, 63 ± 15 and 62 ± 20 μmol/L (*p* < 0.01). Especially, the SUAs of PCW groups were even lower than that of allopurinol control (*p* < 0.01) and were approaching that of the normal control (*p*> 0.05).

**Table 2.** Effects of PCE and PCW on SUA, UUA, BUN and SCr levels and ALT, AST and ALP activities.

Group^a^	Dose (mg/kg)	SUA (µmol)	UUA (mmol)	SCr (µmol)	BUN (mmol)	ALT (U/L)	AST (U/L)	ALP (U/L)
Normal control	Vehicle	71 ± 13	1.1 ± 0.2	34.0 ± 4.9	2.3 ± 0.4	19.5 ± 3.3	65.8 ± 19.6	214.9 ± 42.7
Hyperuricemic control	Vehicle	526 ± 112**	7.0 ± 2.4**	40.8 ± 4.1*	3.7 ± 0.8*	22.9 ± 3.3*	71.9 ± 9.9	197.0 ± 67.6
Allopurinol	5	296 ± 96^##^	4.1 ± 1.0*,#	62.8 ± 5.1^**,##^	9.8 ± 2.6^**,##^	33.0 ± 2.6^**,##^	124.3 ± 24.3^**,##^	267.5 ± 45.4*
Benzbromarone	7.8	258 ± 65^##^	5.9 ± 1.0^**,#^	54.0 ± 4.3^**,##^	5.2 ± 1.5^**,△△^	30.4 ± 2.6^**,##^	93.4 ± 14.8△	207.0 ± 27.3△
PCE	50	178 ± 53^##,△^	5.3 ± 3.8**	43.4 ± 6.0^△△^	4.0 ± 0.5^△△^	24.8 ± 6.1^△△^	90.1 ± 22.4△	193.4 ± 45.8△
	100	153 ± 57^##,△△^	5.1 ± 2.2**	45.3 ± 8.3^△△^	4.2 ± 1.3^△△^	25.8 ± 4.7^△△^	84.9 ± 12.2△	200.6 ± 51.7△
	200	151 ± 62^##,△△^	6.3 ± 2.4**	36.8 ± 9.1^△△^	4.0 ± 1.7^△△^	22.8 ± 6.1^△△^	76.7 ± 34.8^△△^	151.0 ± 69.8^△△^
PCW	50	69 ± 23^ ##,△△ ^	2.2 ± 1.2^##^	27.0 ± 9.3^△△^	2.4 ± 0.9^△△^	16.2 ± 5.7^△△^	43.0 ± 12.3^△△^	100.8 ± 42.5^△△^
	100	63 ± 15^ ##,△△^	2.3 ± 1.3^##^	28.7 ± 5.8^△△^	3.2 ± 2.2^△△^	14.9 ± 2.1^△△^	52.0 ± 12.0^△△^	104.7 ± 12.1^△△^
	200	62 ± 20^ ##,△△^	1.8 ± 0.9^##^	29.8 ± 16.0^△△^	2.2 ± 1.1^△△^	17.5 ± 8.6^△△^	58.4 ± 47.6^△△^	109.9 ± 54.1^△△^

SUA: serum uric acid; UUA: urine uric acid; SCr: serum creatinine; BUN: blood urea nitrogen.

*p< 0.05, **p< 0.01 versus the normal control.

^#^*p*< 0.05, ^##^*p*< 0.01 versus the hyperuricemic control.

^△^*p*< 0.01, ^△△^*p*< 0.01 compared with the allopurinol control.

^a^ *n* = 8.

Since *P. cocos* is a well-known diuretic agent according to the records in Chinese herbal classics, we assayed the UUA to characterize its uricosuric effect. The high-dosed PO and HX for model establishment induced a remarkable rise in UUA in HUA control (7.0 ± 2.4 mmol/L, *p* < 0.01, Table 2) when it was compared with the normal control (1.1 ± 0.2 mmol/L). PCE (5.3 ± 3.8, 5.1 ± 2.2 and 6.3 ± 2.4 mmol/L, *p*> 0.05) did not show significant differences from that of HUA control. However, PCW at various doses showed lower UUAs (2.2 ± 1.2, 2.3 ± 1.3 and 1.8 ± 0.9, *p* < 0.01) than HUA control. These uricosuric effects of reducing UUA may be interpreted by the balance of uric acid production and excretion.

To evaluate the impact of *P. cocos* on kidney function, the serum biochemical parameters related to renal function were determined. The results of SCrs and BUNs showed some correlated features (Table 2). In terms of SCrs, the HUA control (40.8 ± 4.1 μmol/L) showed elevated SCrs as compared to the normal control (34.0 ± 4.9 μmol/L, *p* < 0.05). The SCrs surged further to 62.8 ± 5.1 and 54.0 ± 4.3 μmol/L (*p* < 0.01) in allopurinol and benzbromarone controls. PCE and PCW at various doses presented SCrs at 43.4 ± 6.0, 45.3 ± 8.3 and 36.8 ± 9.1 and at 27.0 ± 9.3, 28.7 ± 5.8 and 29.8 ± 16.0 μmol/L, respectively. PCE at 50 and 100 mg/kg showed no significant SCrs (*p*> 0.05) in comparison to HUA control. However, PCE at high dose and all of PCW groups depicted decreased SCrs significantly (*p* < 0.05).

In comparison to the normal control (2.3 ± 0.4 mmol/L, Table 2), PO and HX in HUA control increased the BUNs slightly (3.7 ± 0.8 mmol/L, *p* < 0.05). Then, allopurinol (9.8 ± 2.6 mmol/L, *p* < 0.01) and benzbromarone (5.2 ± 1.5 mmol/L, *p* < 0.01) further increased BUNs. PCE only heightened BUNs a little to 4.0 ± 0.5, 4.2 ± 1.3, 4.0 ± 1.7 mmol/L (*p*> 0.05) with no significance in comparison with HUA control. Apparently, PCW groups presented BUNs at 2.4 ± 0.9, 3.2 ± 2.2 and 2.2 ± 1.1 mmol/L (*p*> 0.05), which were at the level of normal control. It suggested that *P. cocos* may protect the kidney function in hyperuricemic mice.

Clinically, ALT, AST and ALP activities are commonly used to evaluate hepatic healthy. Hence, we detected ALT, AST and ALP activities to examine the effect of *P. cocos* on liver function (Table 2). HX and PO in HUA control did not induce significant alters on ALT and AST activities (*p*> 0.05). HX and PO declined the ALP activity slightly with no significance (*p*> 0.05). However, allopurinol caused serious liver damage apparently, lifting ALT (*p* < 0.01), AST (*p* < 0.01) and ALP (*p* < 0.05) significantly in comparison to the HUA control. In contrast to the hepatic toxic allopurinol, PCE and PCW presented no negative effects on ALT, AST and ALP activities.

### Inner organ coefficients and morphology changes suggested the nephroprotective and liver protective effects of *P. cocos* extracts

Table 3 shows the effects of *P. cocos* on the body weights. High-dosed PO and HX in HUA control (30.5 + 1.8 g on the 7th) suppressed the weight growth in comparison to normal control (34.2 + 2.8 g on the 7th, *p* < 0.01). Moreover, allopurinol stimulated a significant reduction in body weight (24.1 + 2.1 g on 7th day, *p* < 0.01) in hyperuricemic mice (*p* < 0.01). In the PCE and PCW groups, there were no significant alterations on body weight (*p*> 0.05) when compared to that of the HUA control.

**Table 3.** Body weights of mice on the 1st and 7th days (a) and the organ coefficients of liver (b), kidney (c), spleen (d) and thymus (e) on 7th day.

Group^a^	Dose (mg/kg)	Body weight (g)		Inner organ coefficient (%)			
		1st day	7th day	Liver	Kidney	Spleen	Thymus
Normal control	Vehicle	22.3 + 1.4	34.2 + 2.8	5.4 + 0.1	1.22 + 0.08	0.52 + 0.07	0.42 + 0.05
Hyperuricemic control	Vehicle	22.7 + 1.3	30.5 + 1.8**	5.1 + 0.1	1.81 + 0.15**	0.55 + 0.06	0.37 + 0.05*
Allopurinol	5	22.3 + 0.7	24.1 + 2.1^##^	5.4 + 0.2	1.73 + 0.07**	0.42 + 0.05*,#	0.34 + 0.04**
Benzbromarone	7.8	22.5 + 1.2	28.2 + 2.5	5.2 + 0.2	1.94 + 0.10**	0.44 + 0.06#	0.36 + 0.04*
PCE	50	23.0 + 1.2	30.2 + 3.0^△△^	5.2 + 0.2	1.54 + 0.35#	0.50 + 0.06	0.38 + 0.06
	100	22.5 + 1.3	29.4 + 2.7^△△^	5.2 + 0.5	1.50 + 0.27#	0.49 + 0.06	0.39 + 0.03△
	200	23.3 + 1.6	31.1 + 2.1^△△^	5.1 + 0.1	1.51 + 0.26#	0.54 + 0.08^△△^	0.37 + 0.05
PCW	50	22.0 + 0.9	27.9 + 2.9^△△^	5.1 + 0.2	1.37 + 0.32^##^	0.53 + 0.1^△△^	0.35 + 0.03**
	100	22.9 + 1.3	29.6 + 2.9^△△^	5.4 + 0.3	1.54 + 0.32#	0.56 + 0.04^△△^	0.38 + 0.04
	200	22.6 + 1.8	29.0 + 2.7^△△^	5.3 + 0.3	1.38 + 0.15^##^	0.52 + 0.11△	0.42 + 0.03^△△^

*p< 0.05, **p< 0.01 versus the normal control.

^#^*p*< 0.05, ^##^*p*< 0.01 versus the hyperuricemic control.

^△^*p*< 0.01, ^△△^*p*< 0.01 compared with the allopurinol control.

^a^ *n* = 8.

**Figure 1.**
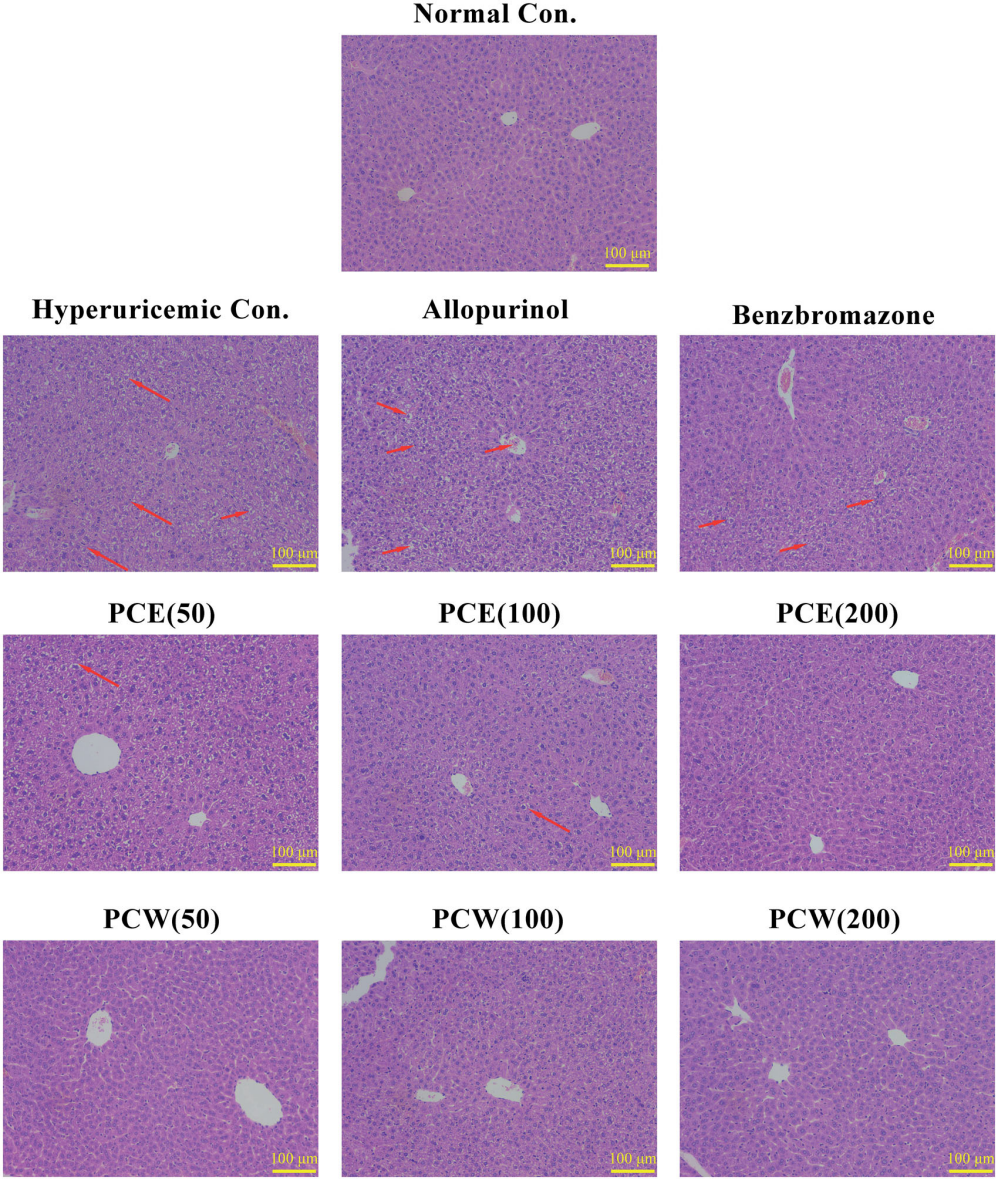
Histological micrographs of liver tissues stained with H&E: normal control, hyperuricemic control, allopurinol control (5 mg/kg), benzbromarzone control (7.8 mg/kg), PCE (50 mg/kg), PCE (100 mg/kg), PCE (200 mg/kg), PCW (50 mg/kg), PCW (100 mg/kg) and PCW (200 mg/kg). Magnification, ×200 ×; scale bar, 100 μm; red arrow, necrotic and/or inflammatory foci.

Since inner organs played important roles in urate homeostasis, we examined the influence of *P. cocos* on inner organ coefficients for further assessment of the health of inner organs under PCE and PCW influences. Table 3 provides the liver coefficients, where no significant difference between hyperuricemic and normal controls was observed. Also, the liver coefficients of PCE and PCW groups showed no significant difference when they were compared to that of the HUA control. Even when they were compared to the normal control, PCE and PCW groups displayed no significant change in liver coefficients. These may demonstrate that PCE and PCW may exert no negative effect on liver function. The HUA control (1.81 ± 0.15%) showed an increased kidney coefficient than that of the normal control (1.22 ± 0.08%, *p* < 0.01), suggesting negative influences of HUA on renal function. Allopurinol (1.73 ± 0.07%) and benzbromarone (1.94 ± 0.10%) controls maintained the high kidney coefficient of the HUA control (*p*> 0.05). It was worth to notice that PCE and PCW at various doses exhibited the declined kidney coefficients at 1.54 ± 0.35%, 1.50 ± 0.27%, 1.51 ± 0.26%, 1.37 ± 0.32%, 1.54 ± 0.32% and 1.38 ± 0.15% in hyperuricemic mice (*p* < 0.05). These meant that PCE and PCW could attenuate the renal injuries caused by HUA. As for spleen coefficients in Figure 1(d), HUA control just exhibited a small increase (0.55 ± 0.06%, *p*> 0.05) in that when it was compared with the normal control (0.52 ± 0.07%). Allopurinol (0.42 ± 0.05%, *p* < 0.01) and benzbromarone (0.44 ± 0.07%, *p* < 0.05) caused spleen strinkage. PCE and PCW showed spleen coefficients at 0.50 ± 0.06%, 0.49 ± 0.06%, 0.54 ± 0.08%, 0.53 ± 0.10%, 0.56 ± 0.04% and 0.52 ± 0.11% (*p* > 0.05) and no significant difference was observed when they were compared to HUA control, suggesting that it exerted no obvious impact on spleen. However, the HUA control produced a negligent reduction in thymus index (0.37 ± 0.05%, *p* < 0.05) in comparison to the normal control (0.42 ± 0.05%, Figure 1(e)). Different from the further deterioration by allopurinol (0.34 ± 0.04%, *p* < 0.01) and benzbromarone (0.36 ± 0.04%, *p* < 0.05) in hyperuricemic mice, PCE at 50, 100, 200 mg/kg and PCW at 100, 200 mg/kg showed thymus coefficients at 0.38 ± 0.06%, 0.39 ± 0.03%, 0.37 ± 0.05%, 0.38 ± 0.04% and 0.42 ± 0.03% (*p*> 0.05), which were approximating to that of the normal control.

**Figure 2.**
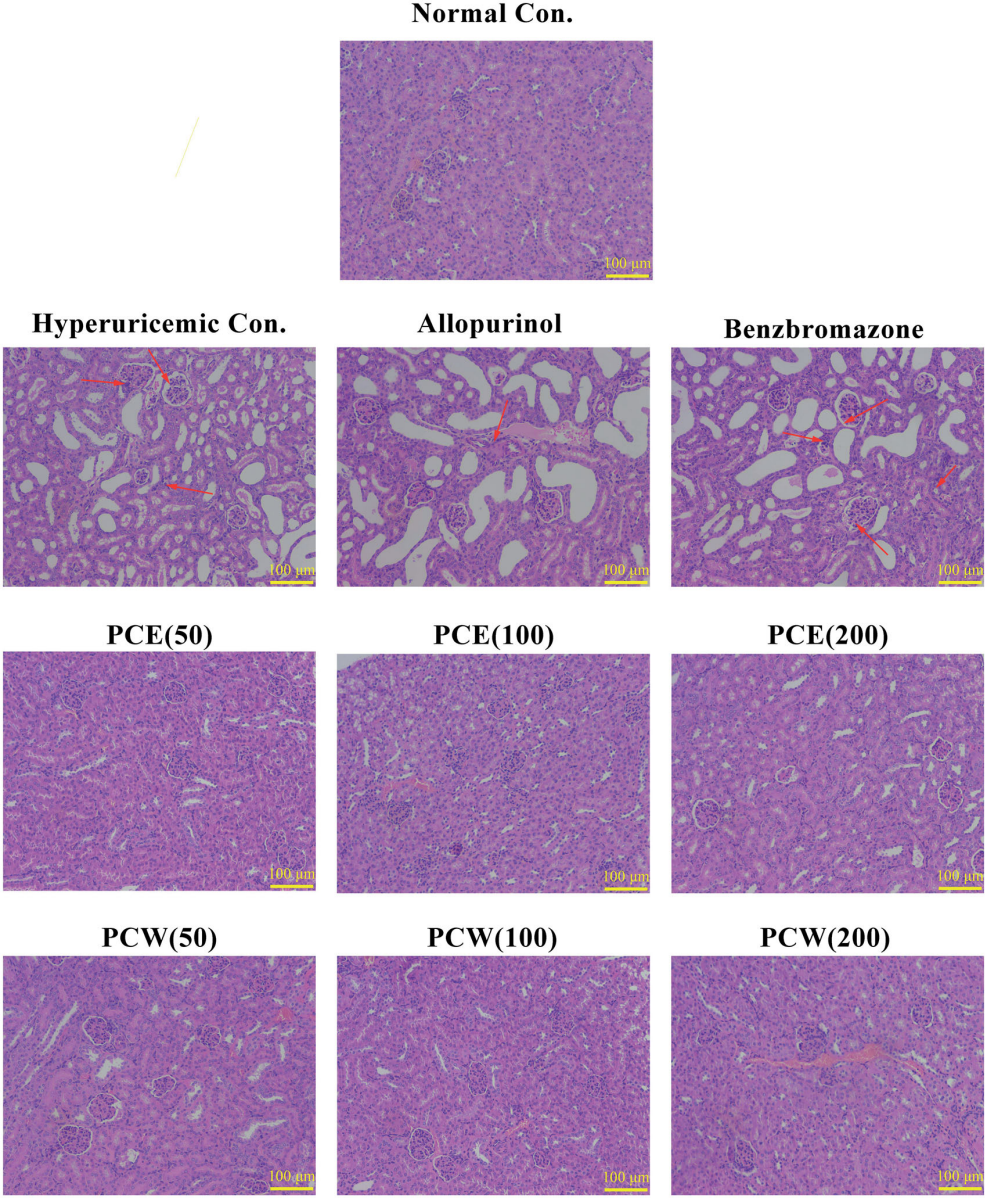
Histological micrographs of kidney tissues stained with H&E: normal control, hyperuricemic control, allopurinol control (5 mg/kg), benzbromarzone control (7.8 mg/kg), PCE (50 mg/kg), PCE (100 mg/kg), PCE (200 mg/kg), PCW (50 mg/kg), PCW (100 mg/kg) and PCW (200 mg/kg). Magnification, ×200 ×; scale bar, 100 μm; red arrow, necrotic and/or inflammatory foci.

Since kidney functions for urate transportation, the histological examinations of kidneys were performed (Figure 2). Compared with the normal control, the HUA control demonstrated morphological changes in renal tissues as following: the brush border of epithelial cell disappeared, glomerular hypertrophy increased, the mesangial areas were widened, the renal tubules were shrunk and lumen dilated. The degree of renal damage was increased and basic renal architectures were destroyed further by allopurinol and benzbromarone (such as for glomerulus and renal tubules) in comparison to the HUA control. Among the renal interstitium in the HUA, allopurinol and benzbromarone controls, the inflammation response accompanied with abundant lymphocytes within the mixed cellular infiltrate was found. PCE and PCW at various doses attenuated the kidney damages, restored renal architectures and ameliorated the inflammatory cell infiltration in renal interstitium caused by HUA.

Since liver plays a central role in uric acid production, the histological examinations of liver tissues were performed (Figure 1). Liver tissues of mice treated with HX and PO in HUA control developed slight hepatic damages. As compared to normal controls, the HUA control was characterized with increased Kupffer cells and inflammatory corpuscles in the cytoplasm of swelled hepatic cells and blurred boundary of hepatic lobule. Allopurinol deepened the damage features of hepatic tissues further. Also, some damaging features were shown in benzbromarone control. Importantly, the inflammatory response caused by PO and HX was alleviated by PCE. PCW group showed clear hepatic lobules and regular hepatocytes, similar to the normal group. Thus, by administrating *P. cocos* extracts, liver damage in hyperuricemic mice were ameliorated.

### Up-regulation of ABCG2, OAT1, OAT3 and OCT2 by *P. cocos* extracts

**Figure 3.**
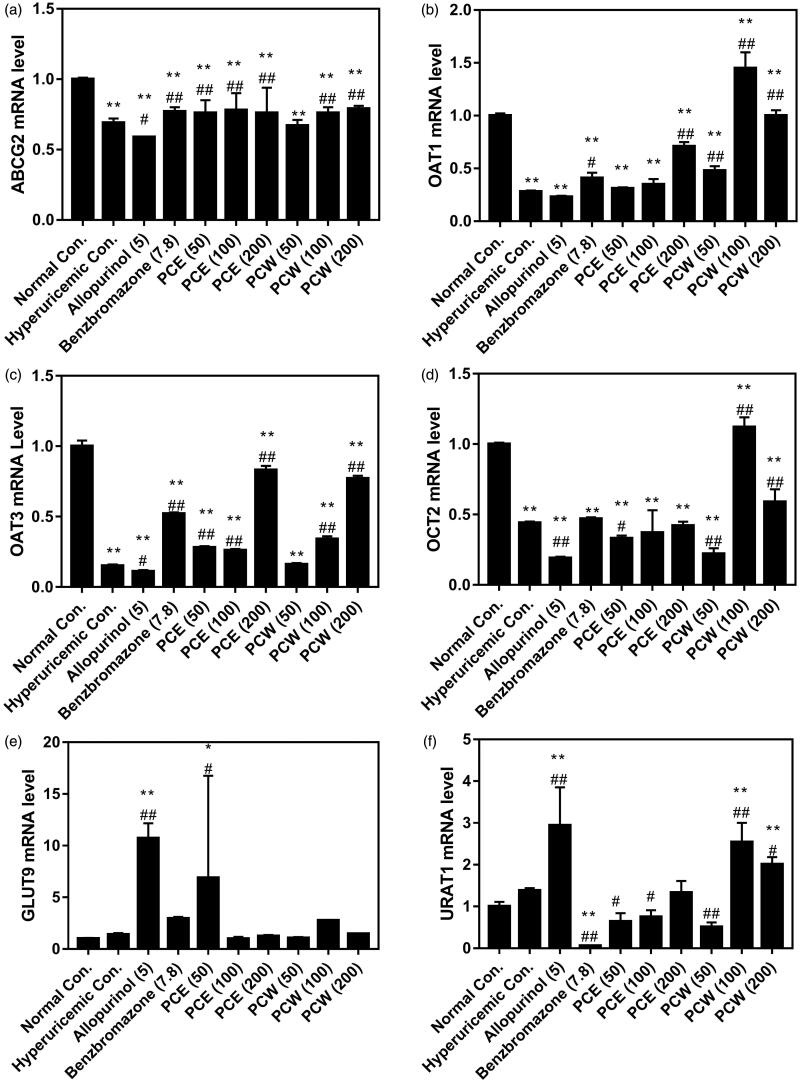
Effects of PCE and PCW on renal ABCG2 (a), OAT1 (b), OAT3 (c), OCT2 (d), GLUT9 (e) and URAT1 (f) mRNA expression. *n* = 3. * *p* < 0.05, ** *p* < 0.01 versus the normal control; ^#^ *p* < 0.05, ^##^ *p* < 0.01 versus the hyperuricemic control.

The renal key mRNA expressions of ABCG2, OAT1, OAT3, OCT2, GLUT9 and URAT1 in hyperuricemic mice with different treatments are depicted in Figure 3. High-dosed HX and PO as HUA inducers in the HUA control suppressed ABCG2, OAT1, OAT3 and OCT2 mRNA (*p* < 0.01, Figure 3(a–d)) expression in comparison to the normal control. However, PCE and PCW ameliorated mRNA decreases of ABCG2, OAT1, OAT3 and OCT2. Specifically, GLUT9 and URAT1 mRNA were lift slightly (*p*> 0.05) by PO and HX but they were not down-regulated by PCE and PCW (Figure 3(e,g)).

**Figure 4.**
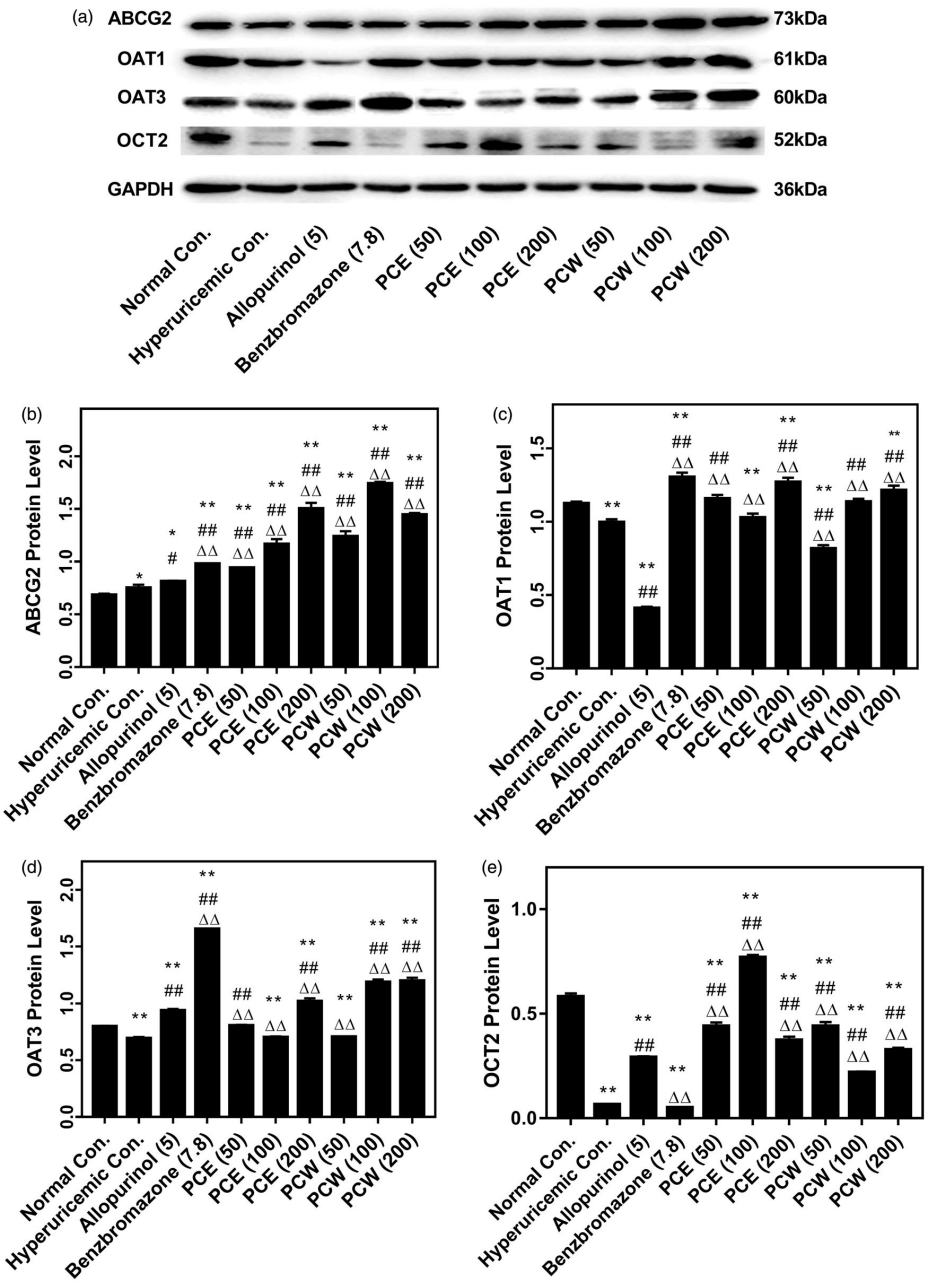
Effects of PCE and PCW on renal ABCG2, OAT3, OAT1 and OCT2 protein expression detected by Western blot: immunoreactive bands (a) and densitometries (b,c,d and –e, expressed as mean ± SD; *n* n = 3). *p < 0.05, **p < 0.01 versus the normal control; ^#^*p* < 0.05, ^##^*p* < 0.01 versus the hyperuricemic control; ^△△^*p* < 0.01 versus the allopurinol control.

The influences of PCE and PCW on key kidney protein expression of ABCG2, OAT1, OAT3 and OCT2 in hyperuricemic mice were investigated (Figure 4). The western blotting bands are shown in Figure 4(a), depicting that HX and PO remarkably suppressed OAT1, OAT3 and OCT2 protein expressions (*p* < 0.01). The up-regulated ABCG2 protein expressions were observed significantly in the HUA mice treated with benzbromarone, PCE and PCW (*p* < 0.01, Figure 4(b)). Specifically, the promoting effects of PCE and PCW on ABCG2 were even better than that of the benzbromarone control. In terms of OAT1 and OAT3 protein, its targeting drug, benzbromarone, showed a strong promotion effect (*p* < 0.01, Figure 4(c,d)). PCE and PCW provoked obvious increases on OAT1 protein expressions (*p* < 0.01) and their up-regulation effects at the high doses were approximating to that of the benzbromarone group. PCE and PCW at high doses up-regulated OAT3 protein expressions significantly (*p* < 0.01). In terms of OCT2 protein expression in Figure 4(e), PCE and PCW up-regulated its expressions (*p* < 0.01), which was significantly suppressed in the HUA control.

### Potential bioactives against hyperuricaemia interacted with ABCG2 molecular docking using the in-house database of *P. cocos*

**Figure 5.**
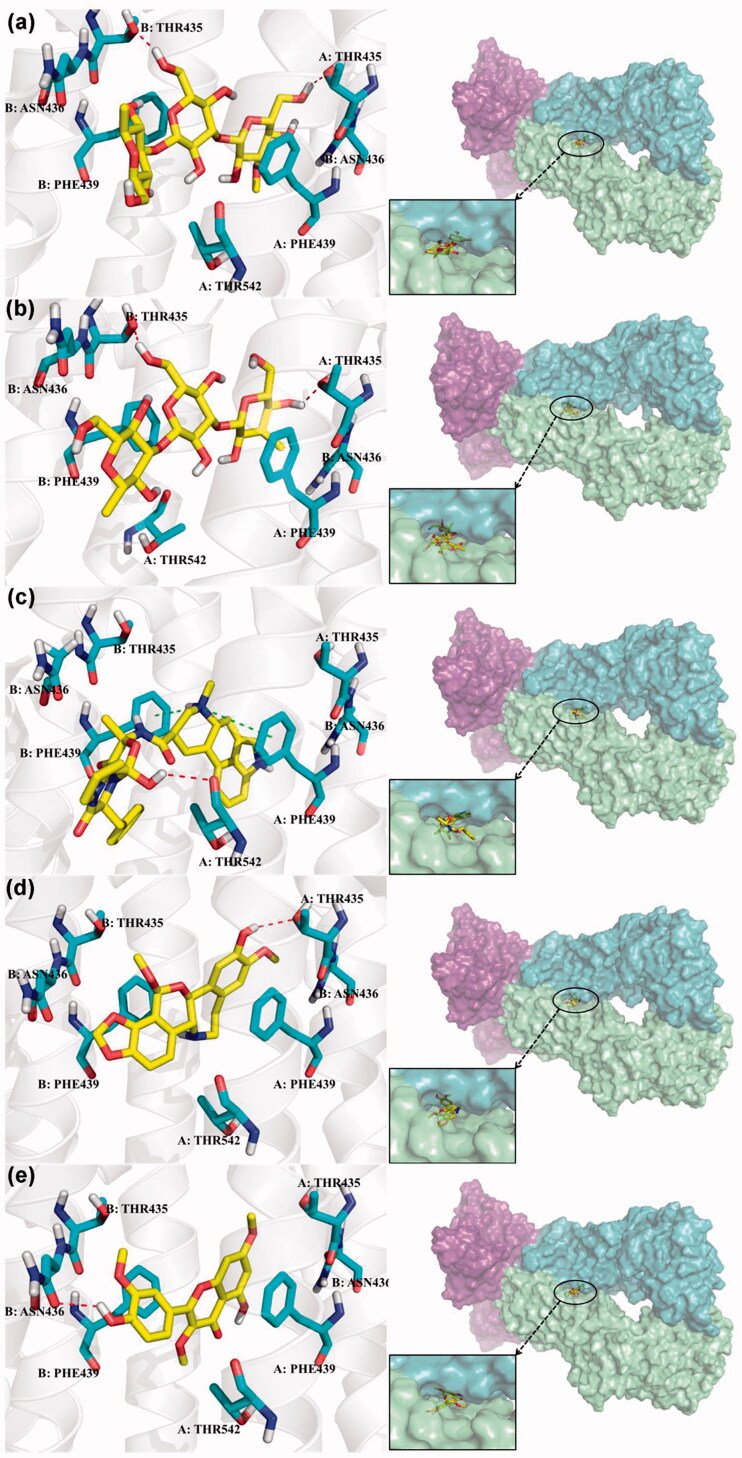
Binding modes of the top five ranked compounds to ABCG2: Cpd267 (a), Cpd277 (b), Cpd13824 (c), Cpd15730 (d) and Cpd5759 (e). The dashed lines represent hydrogen bond or Pi-Cat stacking.

Based on the notable ABCG2 up-regulations by PCE and PCW, we selected an ABCG2 structure of high-resolution as the receptor for molecular docking to screen the hypouricaemic bioactives in *P. cocos*. Five top-ranking compounds were selected for detailed analysis (Table 4). The top-five compounds had binding energies and interaction energies even better than the positive compound, oestrone 3-sulfate (E1S) (Jackson et al. 2018). Hydrogen bonds were involved in the orientations and interactions of each compound to the ABCG2 receptor. Five candidates, presented in Figure 5, compounds **267**, **277**, **13824**, **15730** and **5759** generated hydrogen bonds with key residues of THR435, ASN436 and THR 542. Among them, amine moiety of **13824** provoked Pi-Cat stacking with PHE439 on A and B chains, respectively. All candidates were bound with ABCG2 in the substrate-binding site, which may promote transfer function of ABCG2.

**Table 4.** Screened compounds and structures from *P. cocos* for ABCG2 by molecular docking, wherein the *in situ* compound E1S in ABCG2 served as positive control.

Compound	Structure	Database source	Interaction energy	CDOCKER energy
Cpd267	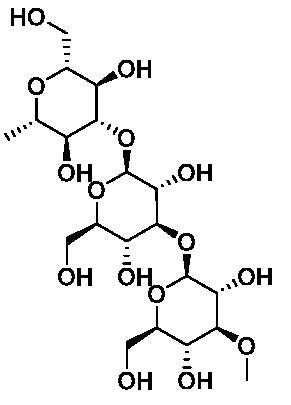	TCMSP	61.591	16.105
Cpd277	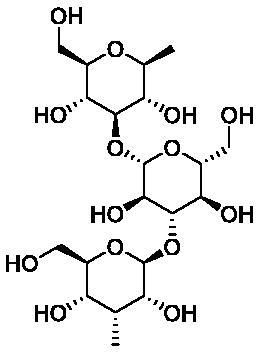	TCMSP	57.353	12.873
Cpd13824	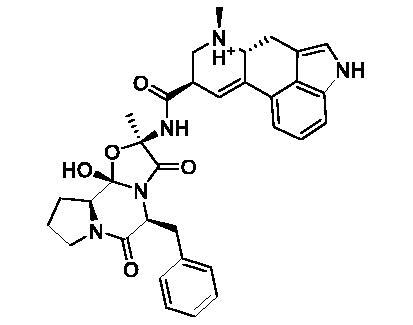	TCMID	54.107	3.141
Cpd15730	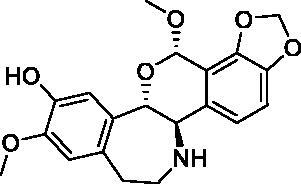	TCMID	44.932	12.196
Cpd5759	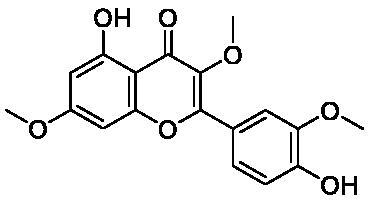	TCMID	39.074	23.386
E1S	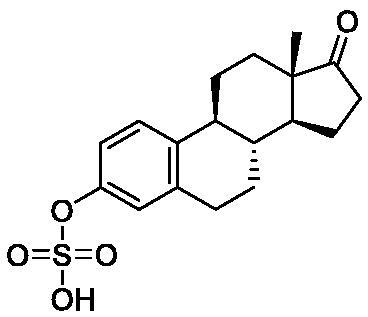	pubchem	33.298	4.767

## Discussion

According to the records in TCM classics for *P. cocos* of ‘eliminating dampness’, ‘promoting diuresis’ and ‘invigorating the spleen’, we hypothesized that *P. cocos* may exert hypouricaemic effect via promoting urate excretion. Herein, we investigated the hypouricaemic effect of *P. cocos* by administrating the ethanol and water extracts to hyperuricemic models, which were established by dosing PO and HX. One unanticipated finding was that it showed some nephron protective effect. The pharmacological activity against HUA was supported by its regulation of the key targets, including renal ABCG2, OAT1, OAT3 and OCT2, especially ABCG2. Also, compound database of *P. cocos* was established and a screening of potential active compounds against HUA in *P. cocos* was performed *in silico*.

PO and HX are frequently employed to establish HUA animal models for researching HUA. In the present study, in combination, PO and HX induced HUA, which was confirmed by a dramatic increase of the SUAs (Taylor et al. 2017) in HUA control and also this success was reaffirmed by the practical decreases of SUAs by allopurinol and benzbromarone in positive controls. In this model, the hypouricaemic effects of *P. cocos* extracts were investigated and then it demonstrated that *P. cocos* was even more effective than positive allopurinol and benzbromarone controls. Meanwhile, dosing with PO and HX leaded to remarkably elevated UUA. Considering the SUA result with the UUA result together, PCE and PCW may evoke uricosuric effect in HUA mice since there were expanded differences between SUA and UUA.

SCr and BUN, as critical indicators of renal functions (Liang et al. 2018), usually surge when renal function is impaired. PO and HX caused some renal damage indicated by the surges of SCrs and BUNs, which then were lowered by PCE and PCW subsequently. In contrast to the evident nephrotoxicity of allopurinol and benzbromarone (Lee et al. 2008; Bouchard et al. 2009), *P. cocos* extracts did not elicit renal toxicity; and, in contrast, it showed some nephroprotective effects. ALT, AST and ALP are considered as critical indicators for hepatic function and they were generally increased when liver functions were injured (Altınok-Yipel et al. 2020). Compared to noticeable elevations of ALT, AST and ALP activities in the allopurinol group, the PCE and PCW showed no negative effects on them. Moreover, histopathological analysis supported some protective or repair effects on kidney and liver by PCE and PCW. This was consistent to the results of biochemical parameters and inner organ coefficients.

To elucidate the mechanisms underlying the hypouricaemic action of *P. cocos*, we tested the effects on the main renal transporters (Li JM et al. 2011) since its urate excretion was implied in TCM classics. Renal transporters, including ABCG2, OAT1, OAT3, OCT2, GLUT9 and URAT1, are directly associated with the homeostasis of SUA. Among them, ABCG2 is an efficient urate exporter, the dysfunction of which raises gout/HUA risk via extra-renal pathway (Sakurai 2013; Matsuo et al. 2014). PCE and PCW increased the mRNA and protein expressions of ABCG2, inducing the remarkably enhanced uric acid excretion by advancing the uric acid transportation through ABCG2. PO and HX were used to establish hyperuricemic models by down-regulating mRNA and proteins of OAT1, OAT3 and OCT2, which were organic ion transporters laid on the basolateral of renal cells, functioning for urate transportation (Ichida et al. 2012). Herein, these were alleviated by PCE and PCW. But PCE and PCW did not decrease URAT1 or GLUT9. All above, the hypouricaemic effect of *P. cocos* may be raised by up-regulation of ABCG2, OAT1, OAT3 and OCT2 rather than influencing URAT1 and GLUT9.

To screen the bioactives of the hypouricaemic action of *P. cocos* against HUA, molecular docking was conducted. Five top-ranked candidates were selected for analysis. All of them were located at the entrance tunnel for substrate to catalytic centre, probably stimulates the ATPase activity of the transporter by stabilizing the transmembrane domains interface (Jackson et al. 2018). Hydrogen bond and Pi-cation interactions played important roles for compound orientations and locations. Further investigations may be performed to obtain and investigate these screened bioactive *in vitro* and *in vivo.*

## Conclusions

We reported the hypouricaemic and nephroprotective effects of *P. cocos* in hyperuricemic mice. The hypouricaemic effect of *P. cocos* may be attributed to its up-regulation on renal ABCG2. Since its key role is on ABCG2, we chose this as the target for virtually screening the bioactives against HUA by molecular docking and five compounds were found with high ranks. In the future, the screened bioactives should be examined. These results may be useful for the development of a hypouricaemic agent and even the drug discovery against HUA from *P. cocos*.

## Supplementary Material

Supplemental Material
