# Supplementary material for: Hypouricaemic and nephroprotective effects of Poria cocos in hyperuricemic mice by up-regulating ATP-binding cassette super-family G member 2
Source: Pharm Biol. 2021 Mar 2;59(1):273–84. doi: 10.1080/13880209.2021.1885450 (PMC7928048; doi:10.1080/13880209.2021.1885450)
Supplement: Supplemental Material [file IPHB_A_1885450_SM1372.doc]

**Submission to Pharmaceutical Biology**

**Supplementary Material**

**The following is included as additional supporting materials for this paper:**

**Experimental**

***HPLC conditions***

Chromatography was performed on a reversed-phase column (Waters Atlantis T3 RP-C18 column, 5μm, 250 mm × 4.6 mm) with a flow rate of 1 ml/min. Separation was carried out by programed gradient elution with acetonitrile (B) and 0.1 % phosphoric acid aqueous solution (A) as following: 50~55%, 0~30 min, B; 55~60%, 30~40 min, B; 60~65%, 40~50 min, B; 65~70%, 50~60 min, B; 70~75%, 60~70 min, B; 75~85%, 70~80 min, B; 85~90%, 80~85 min, B; 90%, 85~90 min, B. The detection wavelength was set at 210 nm, and the column temperature was kept at 30 ^o^C. The loading volume was 10 μl.

**Figure. S1** The fingerprint of PCE. HPLC conditions-column: Waters Atlantis T3 RP-C18 column, 5μm, 250 mm × 4.6 mm; flowing rate: 1 ml/min; detection wavelength: 210 nm; temperature: 30 ^o^C; injection: 10 μL.

**Figure. S2** The fingerprint of PCW. HPLC conditions-column: Waters Atlantis T3 RP-C18 column, 5μm, 250 mm × 4.6 mm; flowing rate: 1 ml/min; detection wavelength: 210 nm; temperature: 30 ^o^C; injection: 10 μL.

**Figure. S3** A HPLC chromatogram of the standard chemicals (pachymic acid) for *P. cocos* identification. HPLC conditions-column: Waters Atlantis T3 RP-C18 column, 5μm, 250 mm × 4.6 mm; flowing rate: 1 ml/min; detection wavelength: 210 nm; temperature: 30 ^o^C; injection: 10 μL.
